# Effect of Different Clarification Methods on Volatile Aroma Compound Composition of Virgin Olive Oil

**DOI:** 10.17113/ftb.57.04.19.6401

**Published:** 2019-12

**Authors:** Karolina Brkić Bubola, Marina Lukić, Igor Lukić, Olivera Koprivnjak

**Affiliations:** 1 Institute of Agriculture and Tourism, K. Huguesa 8, 52440 Poreč, Croatia; 2 University of Rijeka, Faculty of Medicine, Braće Branchetta 20, 51000 Rijeka, Croatia

**Keywords:** industrial filtration, natural sedimentation and decantation, virgin olive oil, volatile compounds, sensory profile

## Abstract

This study investigates the effect of industrial scale filtration of fresh monovarietal virgin olive oil from Buža and Istarska bjelica cultivars on their volatiles, total phenols and sensory characteristics, and compares the oil samples clarified by filtration with those clarified by natural sedimentation/decantation after six months of storage. Filtration had a different effect on volatiles from the oil samples obtained from different cultivars. In the oil from Buža cultivar immediately after filtration only the amount of (*Z*)-2-pentenol slightly increased, but in Istarska bjelica the oil filtration affected eight compounds (the amount of hexanal, (*E*)-2-pentenal, (*Z*)-3-hexenal, (*Z*)-2-pentenol and (*Z*)-3-hexen-1-ol increased, while of hexyl acetate, (*E*)-2-penten-1-ol and (*E*)-2-hexen-1-ol decreased). In fresh filtered oil from Buža cultivar a slight decrease of total phenols was observed, while in those from Istarska bjelica the decrease was sharp, causing a decrease in the pungency and bitterness. Sedimentation/decantation had advantages over oil filtration of both cultivars, due to improved effect on the preservation of the sensory profile and the level of total phenols. Tentative aroma profiles based on odorant series obtained from the odour activity values were compared to the actual olive oil sensory profiles. These results could have a high level of applications in the olive oil industry for the optimization of the technology for obtaining monovarietal virgin olive oil with preserved specific and typical sensory characteristics, but also may serve experts to choose an appropriate virgin olive oil clarification method prior to analysis of volatile compounds.

## INTRODUCTION

Specific odour and taste of virgin olive oil (VOO) originate from its minor components, particularly volatile and phenolic compounds (*1*). The most important volatiles that contribute to the particular aroma are C6 and C5 derived through the lipoxygenase pathway during VOO production (*2*). The concentration and activity of enzymes involved in the biogenesis of VOO volatile compounds are influenced by several agronomical and technological factors, such as cultivar (*3*, *4*), stage of olive ripeness and production conditions (*2*, *5*, *6*). During production of monovarietal olive oil, it is very important to choose optimal agronomic and technological conditions to obtain the oil with high quality characteristics but also to preserve its specific sensorial characteristics.

Immediately after extraction, the virgin olive oil contains particles of olive fruit and vegetable water, which could deteriorate its quality (*7*). To stabilize the product prior to bottling and selling on the market and to diminish its sensory deterioration during storage, oil clarification is commonly applied. Traditionally, in small-scale olive oil production, the virgin olive oil is clarified by natural sedimentation combined with oil decantation from the sediment. More recently, in order to hasten product finalization, VOO has been filtered using various types of filtration systems (*8*). Small companies often use a filter press with a cellulose filter aid because of its affordable price (*9*). During VOO clarification, changes of minor components may occur affecting its quality (*8*). Reboredo-Rodríguez *et al*. (*10*) demonstrated that natural sedimentation and decantation may have an effect on volatiles and sensory characteristic of the VOO, but it differs depending on the cultivar. Veneziani *et al*. (*11*) also found that changes in volatile composition of VOO from different geographical origin do not show the same trend after filtration using filter press. Only a few studies investigated the effect of VOO filtration by the filter press on volatile composition, focusing the attention only on the difference between the filtered and unfiltered samples (*11*-*13*).

The objective of this study is to investigate the effect of industrial scale filtration of monovarietal virgin olive oil from Buža and Istarska bjelica cultivars on volatile profile, total phenols and sensory characteristics immediately after production, as well as to compare the oil samples clarified by two different methods, filtration and natural sedimentation/decantation, after six months of storage. Furthermore, this study aims to compare the tentative aroma profiles of the virgin olive oil obtained by grouping the odour activity values of volatile compounds into odorant series with the sensory analysis results obtained by a panel trained for the sensory analysis of virgin olive oil. This approach based on the tentative sensory profiling using odorant series has rarely been used in VOO research up to date (*14*, *15*), although it can provide additional useful information because it enables to relate quantitative volatile compound data to sensory perception (*14*).

To our knowledge, this is the first study that compares the effect of filtration by filter press and natural sedimentation combined with oil decantation on the volatile composition of virgin olive oil, and the first study about the influence of industrial filtration of virgin olive oil from Istarska bjelica and Buža cultivars on their volatile composition. These two olive cultivars are the most economically important autochthonous cultivars in Istrian region, Croatia (*16*), and their monovarietal VOOs are characterized by different sensorial profile and composition (*17*). Istarska bjelica is richer in phenolic compounds and has more robust character with higher intensities of bitterness and pungency than Buža oil. The oil from Istarska bjelica is characterized by a simple green odour (green olive fruits, green grass), while that from Buža has a more complex olive fruitiness accompanied by notes of aromatic herb and almond (*4*, *17*, *18*). In this study, olive fruits of both cultivars with the same ripeness degree and produced under the same VOO production conditions were used to avoid differences caused by these factors.

## MATERIALS AND METHODS

### Preparation of virgin olive oil samples

Monovarietal virgin olive oil (VOO) samples from Buža and Istarska bjelica (from now on in the text referred to as Buža and Istarska bjelica oil) were obtained at the beginning of October 2014 from fruits at the same ripening degree (RI=2; (*19*)) from the olive orchard located in Vodnjan, Istria region (Croatia) using two-phase centrifugal system (model SPI 222 S; Pieralisi, Jesi (Ancona), Italy) consisting of a knife crusher, malaxer (35 min at (25±1) °C) and two-phase centrifugal decanter. After the two-phase decanting, the oil samples were filtered immediately or put through a natural sedimentation process to remove the remaining fruit particles and vegetable water. Filtration process was done in triplicate per each monovarietal oil using cellulose filter press system (Euro 20; MORI-TEM, Tavarnelle Val di Pesa, Italy) accompanied by 19 pieces of cellulose filter plates (OV110; Omniafiltra, Alife, Italy) using the same processing conditions (*p*=1.5 kPa, *t*=20 °C). Unfiltered and filtered monovarietal VOO samples were stored into 1-litre opaque glass bottles. Analysis of three bottles per treatment of each cultivar was done immediately after oil production. In order to compare two different clarification methods, three bottles of each unfiltered monovarietal oil were left to settle down naturally for 45 days at 16 °C, and then the oil was decanted from the sediment and transferred into other clean bottles and stored for six months at 16 °C. Filtered and decanted monovarietal oil samples were additionally analysed after a six-month storage under the same conditions. Considering the basic quality parameters (free fatty acids, peroxide value and the specific absorbance coefficients at wavelengths of 232 and 270 nm: *K*_232 nm_ and *K*_270 nm_, respectively), all samples used in this study were within stipulated limits of the extra virgin olive oil category according to the European Commission Regulation (*20*) (data not shown).

### Sensory analysis

VOO sensory analysis was performed by a panel consisting of eight assessors (5 females and 3 males, average age: 36) trained for VOO sensory analysis according to the International Olive Council’s method described in the European Commission Regulation (*20*). Differently from the standard method, more descriptors to describe fruity odour (olive fruit, green grass and apple) and taste attributes (pungent, bitter and sweet) were used. Furthermore, total sensory score, using nine-point overall rating scale graded with points from 1 (the lowest quality) to 9 (the highest quality) served to compare overall quality of the investigated oil samples.

### Analysis of total phenols

Total phenols were extracted from oil diluted with hexane by liquid-liquid extraction with methanol/water (60:40, *V*/*V*) according to the method of Gutfinger (*21*). The content of total phenols was estimated based on the phenol reaction with the Folin-Ciocalteu reagent and sodium carbonate, and measurement of absorbance at 725 nm with spectrophotometer (Carry UV/Vis 50; Varian Inc., Harbor City, CA, USA). Caffeic acid was used as reference for the calibration curve and expression of results (mg caffeic acid per kg of oil). Hexane of analytical quality, methanol of spectrophotometric grade and caffeic acid (≥98%) were obtained from Sigma-Aldrich, Merck (Steinheim, Germany), and Folin–Ciocalteu reagent and sodium carbonate of analytical quality were obtained from Kemika (Zagreb, Croatia). Grade 2 water was obtained from Elix 3 system (Millipore, Bedford, MA, USA).

### Analysis of volatile compounds

The volatile compounds were extracted by the headspace solid-phase microextraction and analysed by GC (Varian 3350; Varian Inc) and GC-mass spectrometry (Varian 3900 GC coupled to a Varian Saturn 2100T ion trap mass spectrometer; Varian Inc.) according to the method reported by Brkić Bubola *et al*. (*22*). Differently from the above cited method, for the GC analysis of volatile compounds, a longer capillary column Rtx-WAX (60 m×0.25 mm i.d.×0.25 μm film thickness; Restek, Bellefonte, PA, USA) and higher carrier gas pressure at the head of the capillary column (138 kPa) were utilized to obtain better separation of particular volatiles. Volatile compound standards (GC purity ˃95%) used for the identification and quantification of volatiles were purchased from Sigma-Aldrich, Merck, Fluka (Steinheim, Germany) and Alfa Aesar (Karlsruhe, Germany).

### Odour activity values and odorant series

The odour activity values (OAV) of volatile compounds were calculated by dividing the concentration of each compound by its odour threshold concentration from the literature (*23*-*28*) (Table 1). The OAVs of volatiles having similar olfactory sensations were grouped into six odorant series (olive fruit, green grass, apple, sweet, bitter and pungent). The overall intensity of each odorant series was calculated as the sum of the OAVs of all the volatiles associated with this series (*23*-*28*), according to the suggestion by Reboredo-Rodríguez *et al*. (*14*) but with some modifications presented in Table 1.

**Table 1:** Volatile compounds, odour threshold and odorant series used for the quantitative analysis of tentative aromatic profile of virgin olive oil

Volatile compound	Odorant series	Odour threshold*/(ng/g)
3-Methylbutan-1-al	sweet	5.4
	olive fruit	5.4
Ethyl 2-methylbutyrate	olive fruit	0.7
1-Penten-3-on	grass	50
	bitter	0.73
	pungent	0.73
Hexanal	grass	300
	apple	80
	sweet	75
(*E*)-2-pentenal	apple	300
(*Z*)-3-hexenal	grass	1.7
(*E*)-2-hexenal	grass	1125
	apple	424
	bitter	420
Hexyl acetate	grass	1040
	olive fruit	1040
	sweet	1040
(*Z*)-2-pentenol	olive fruit	250
	sweet	250
(*E*)-2-penten-1-ol	bitter	300
(*Z*)-3-hexenyl acetate	grass	750
	olive fruit	750
Hexanol	olive fruit	400
(*E*)-3-hexen-1-ol	bitter	1500
(*Z*)-3-hexen-1-ol	apple	1100
(*E*)-2-hexen-1-ol	grass	8000
(*Z*)-2-hexen-1-ol	grass	1000
*Odour threshold values assessed in refined olive oil and odour descriptors reported in the literature (*23*-*28*)		

### Statistical analysis

Differences among VOO samples were tested by one-way ANOVA at 5% significance level, using Statistica v. 13.2 (*29*). The comparison of mean values was determined with the Tukey’s honestly significant difference test (p˂0.05). Linearity of correlation between sensory intensity and OAV of a single aroma descriptor was tested by adjusted Pearson correlation coefficient (*r*_adj_).

## RESULTS AND DISCUSSION

### The effect of oil filtration

The mass fractions of volatile compounds in Buža and Istarska bjelica oil samples are presented in Table 2 and Table 3, respectively. A significant difference in the mass fraction (in mg/kg) of particular volatile compounds between the two unfiltered monovarietal samples can be observed: about 10 and 25% higher mass fractions of C5 and C6 volatile compounds, respectively, were determined in the oil from Buža than in Istarska bjelica. C6 volatile compounds, biogenerated through the lipoxygenase pathway from linoleic and linolenic acid by endogenous enzymes, are responsible for the green odour of VOO (*30*), while C5 volatile compounds, produced in an additional branch of the lipoxygenase pathway, are also important for its aroma (*31*).

**Table 2:** Volatile composition of Buža (BU) oil samples: fresh unfiltered (UF), fresh filtered (F), stored for six months after filtration (F6) and stored for 6 months after natural sedimentation and decantation (SD6)

Volatile compound	*w*/(mg/kg)			
BU-UF	BU-F	BU-F6	BU-SD6
3-Methylbutan-1-al	(0.01±0.02)	(0.044±0.009)	(0.027±0.001)	(0.024±0.008)
Ethyl 2-methylbutyrate	(0.001±0.000)	(0.001±0.000)	(0.001±0.000)	(0.001±0.000)
1-Penten-3-one	(0.9±0.1)	(1.0±0.2)	(0.76±0.03)	(0.734±0.008)
Hexanal	(2.7±0.2)^ab^	(3.0±0.2)^a^	(2.0±0.4)^ab^	(1.93±0.05)^b^
(*E*)-2-pentenal	(0.076±0.005)	(0.070±0.008)	(0.059±0.004)	(0.08±0.01)
Isoamyl acetate	(0.008±0.002)	(0.009±0.001)	(0.007±0.000)	(0.010±0.001)
*(Z)*-3-hexenal	(1.38±0.01)^a^	(1.4±0.1)^a^	(0.48±0.02)^b^	(1.23±0.03)^a^
(*E*)-2-hexenal	(34.6±4.1)	(32.3±4.0)	(32.8±0.2)	(35.3±1.4)
Hexyl acetate	(0.022±0.001)	(0.019±0.006)	(0.020±0.002)	(0.021±0.001)
Octanal	(0.091±0.007)^b^	(0.142±0.003)^ab^	(0.16±0.02)^a^	(0.14±0.01)^ab^
(*Z*)-2-pentenol	(0.038±0.006)^b^	(0.177±0.008)^a^	(0.18±0.01)^a^	(0.21±0.03)^a^
(*E*)-2-penten-1-ol	(0.19±0.01)^ab^	(0.20±0.02)^a^	(0.141±0.009)^b^	(0.17±0.02)^ab^
(*Z*)-3-hexenyl acetate	(0.083±0.004)^b^	(0.084±0.005)^ab^	(0.100±0.005)^a^	(0.100±0.002)^a^
Hexanol	(0.52±0.04)^b^	(0.46±0.04)^b^	(0.58±0.04)^ab^	(0.8±0.1)^a^
(*E*)-3-hexen-1-ol	(1.62±0.07)	(1.50±0.07)	(1.44±0.06)	(1.8±0.2)
(*Z*)-3-hexen-1-ol	(0.91±0.02)^b^	(0.89±0.04)^b^	(0.976±0.031)^b^	(1.20±0.07)^a^
(*E*)-2-hexen-1-ol	(0.91±0.01)^ab^	(0.87±0.04)^b^	(1.0±0.04)^ab^	(1.04±0.04)^a^
(*Z*)-2-hexen-1-ol	(0.000±0.000)	(0.000±0.000)	(0.000±0.000)	(0.000±0.000)
(*E*)-2-octenal	(0.024±0.002)^b^	(0.023±0.000)^b^	(0.15±0.04)^a^	(0.100±0.002)^a^
C6 aldehydes	(38.6±3.9)	(36.6±3.9)	(35.4±0.5)	(38.5±1.3)
C6 alcohols	(4.0±0.1)^a^	(3.7±0.1)^b^	(4.0±0.2)^ab^	(4.8±0.4)^a^
C6 volatiles	(42.7±4.1)	(40.4±4.0)	(39.4±0.7)	(43.4±1.7)
C5 volatiles	(1.2±0.1)	(1.5±0.2)	(1.132±0.000)	(1.19±0.05)
The results are expressed as mean values of three independent repetitions of clarifying procedure ± SD. Different letters in a row represent significant differences between mean values (Tukey’s test, p<0.05). C6 aldehydes=hexanal+(*E*)-2-hexenal+(*Z*)-3-hexenal, C6 alcohols=hexanol+(*E*)-3-hexen-1-ol+(*Z*)-3-hexen-1-ol+(*E*)-2-hexen-1-ol+(*Z*)-2-hexen-1-ol, C5 volatiles=(*E*)-2-pentenal+(*Z*)-2-pentenol+(*E*)-2-penten-1-ol+1-penten-3-one				

**Table 3:** Volatile composition of Istarska bjelica (IB) oil samples: fresh unfiltered (UF), fresh filtered (F), stored for six months after filtration (F6) and stored for six months after natural sedimentation and decantation (SD6)

Volatile compound	*w*/(mg/kg)			
IB-UF	IB-F	IB-F6	IB-SD6
3-Methylbutan-1-al	(0.12±0.03)	(0.07±0.02)	(0.09±0.02)	(0.13±0.01)
Ethyl 2-methylbutyrate	0.001±0.000	(0.001±0.000)	(0.001±0.000)	(0.002±0.001)
1-Penten-3-one	(0.70±0.03)^ab^	(0.87±0.07)^a^	(0.734±0.008)^ab^	(0.4±0.2)^b^
Hexanal	(1.56±0.01)^b^	(2.9±0.3)^a^	(1.3±0.1)^b^	(1.2±0.3)^b^
(*E*)-2-pentenal	(0.045±0.003)^b^	(0.067±0.006)^a^	(0.048±0.001)^b^	(0.046±0.001)^b^
Isoamyl acetate	(0.006±0.001)^b^	(0.006±0.000)^ab^	(0.007±0.000)^a^	(0.007±0.000)^a^
*(Z)*-3-hexenal	(0.257±0.006)^c^	(1.32±0.09)^a^	(0.76±0.05)^b^	(0.196±0.005)^c^
(*E*)-2-hexenal	(26.55±0.01)	(29.2±3.1)	(29.4±1.6)	(24.3±0.7)
Hexyl acetate	(0.046±0.001)^a^	(0.031±0.001)^b^	(0.024±0.000)^c^	(0.044±0.001)^a^
Octanal	(0.16±0.011)^ab^	(0.137±0.003)^b^	(0.126±0.001)^b^	(0.20±0.02)^a^
(*Z*)-2-pentenol	(0.049±0.003)^c^	(0.21±0.02)^a^	(0.157±0.007)^b^	(0.236±0.001)^a^
(*E*)-2-penten-1-ol	(0.279±0.007)^a^	(0.23±0.02)^b^	(0.133±0.006)^c^	(0.187±0.001)^b^
(*Z*)-3-hexenyl acetate	(0.088±0.001)	(0.090±0.001)	(0.084±0.002)	(0.092±0.004)
Hexanol	(0.61±0.03)	(0.48±0.03)	(0.56±0.05)	(0.69±0.08)
(*E*)-3-hexen-1-ol	(0.82±0.02)	(1.20±0.04)	(1.1±0.1)	(0.8±0.2)
(*Z*)-3-hexen-1-ol	(0.270±0.001)^b^	(0.74±0.04)^a^	(0.71±0.04)^a^	(0.28±0.02)^b^
(*E*)-2-hexen-1-ol	(2.3±0.4)^a^	(1.12±0.08)^b^	(1.0±0.1)^b^	(2.2±0.3)^a^
(*Z*)-2-hexen-1-ol	(0.000±0.000)	(0.000±0.000)	(0.000±0.000)	(0.000±0.000)
(*E*)-2-octenal	(0.015±0.001)^b^	(0.023±0.003)^b^	(0.041±0.007)^ab^	(0.09±0.03)^a^
C6 aldehydes	(28.36±0.03)	(33.4±3.5)	(31.4±1.7)	(25.7±1.0)
C6 alcohols	(4.0±0.4)	(3.5±0.2)	(3.4±0.3)	(4.0±0.6)
C6 volatiles	(32.4±0.4)	(37.1±3.7)	(34.9±2.1)	(29.8±0.4)
C5 volatiles	(1.08±0.04)^ab^	(1.4±0.1)^a^	(0.92±0.04)^b^	(0.8±0.2)^b^

The results are expressed as mean value of three independent repetitions of clarifying procedure ± SD. Different letters in a row represent significant differences between mean values (Tukey’s test, p<0.05). C6 aldehydes=hexanal+(E)-2-hexenal+(Z)-3-hexenal, C6 alcohols=hexanol+(*E*)-3-hexen-1-ol+(*Z*)-3-hexen-1-ol+(*E*)-2-hexen-1-ol+(*Z*)-2-hexen-1-ol, C5 volatiles=(*E*)-2-pentenal+(*Z*)-2-pentenol+(*E*)-2-penten-1-ol+1-penten-3-one

The Buža oil filtration process caused significant changes in a smaller number of volatile compounds than of Istarska bjelica. In Buža oil only (*Z*)-2-pentenol (contributes to olive fruit and sweet odour notes (*28*)) increased significantly compared to the unfiltered variant, with probably an insignificant influence on odour characteristics since OAV of this compound remained below 1 (Table 4). In Istarska bjelica oil there were eight significantly influenced compounds out of 19 taken in consideration, the mass fraction increases of (*Z*)-3-hexenal (contributes to green odour notes (*28*)) and hexanal (contributes to grass and apple odour note (*28*)) being the major changes among C6 compounds, and of (*Z*)-2-pentenol among C5 compounds. In our previous investigation of olive oil filtration by filter paper under laboratory conditions, we also found an increase in the mass fraction of some alcohols and aldehydes (*22*). Increase of certain volatile compound mass fraction in oil headspace after filtration could be linked to the distribution of volatiles between the oily and the aqueous phase. During filtration, humidity is partly removed from oil and the affinity of slightly polar volatile compounds for the oil matrix subjected to filtration is reduced. According to octanol/water partition coefficient (log *P*(oct/wat)) the most hydrophilic among the analysed compounds are (*Z*)-2-pentenol (0.94), (*E*)-2-penten-1-ol (0.94), 1-penten-3-one (1.15) and (*E*)-2-pentenal (1.15), which are expected to be released more easily from filtered than from unfiltered oil. However, the increase of these compounds in filtered samples was not consistent as regards statistical significance and cultivar (Table 2 and Table 3). Moreover, in filtered samples, significant increase of less hydrophilic compounds was noticed, such as (*Z*)-3-hexenal and (*Z*)-3-hexen-1-ol (log *P*(oct/wat)=1.54 and 1.61, respectively) in Buža sample as well as hexanal (log *P*(oct/wat)=1.77) in both monovarietal samples. This finding suggests a more complex release mechanism than simple distribution between the oily and the aqueous phase.

**Table 4:** Odour activity values (OAV) of the main odorant series in Buža (BU) and Istarska bjelica (IB) oil samples: fresh unfiltered (UF), fresh filtered (F), stored for six months after filtration (F6) and stored for six months after natural sedimentation and decantation (SD6)

Odorant series	Volatile compound	OAV								
BU-UF	BU-F	BU-F6	BU-SD6		IB-UF	IB-F	IB-F6	IB-SD6
Olive	(*E*)-2-hexen-1-ol	(0.18±0.00)^ab^	(0.17±0.01)^b^	(0.19±0.01)^ab^	(0.21±0.01)^a^		(0.46±0.08)^a^	(0.22±0.02)^b^	(0.21±0.02)^b^	(0.44±0.06)^a^
fruit	(*Z*)-3-hexen-1-ol	(0.83±0.02)^b^	(0.81±0.03)^b^	(0.89±0.03)^b^	(1.09±0.06)^a^		(0.25±0.00)^b^	(0.68±0.04)^a^	(0.65±0.04)^a^	(0.26±0.02)^b^
	3-Methylbutan-1-al	(1.9±2.8)	(8.1±1.7)	(4.9±0.1)	(4.4±1.4)		(21.3±5.2)	(13.1±2.8)	(15.8±3.0)	(24.8±1.8)
	Ethyl 2-methylbutyrate	(1.43±0.00)	(1.43±0.00)	(1.43±0.00)	(1.43±0.00)		(1.43±0.00)	(1.43±0.00)	(1.43±0.00)	(2.9±1.0)
	Hexyl acetate	(0.02±0.00)	(0.02±0.01)	(0.02±0.00)	(0.02±0.00)		(0.04±0.00)^a^	(0.03±0.00)^b^	(0.02±0.00)^c^	(0.04±0.00)^a^
	(*Z*)-2-pentenol	(0.15±0.03)^b^	(0.71±0.03)^a^	(0.71±0.05)^a^	(0.8±0.1)^a^		(0.20±0.01)^c^	(0.82±0.06)^a^	(0.63±0.03)^b^	(0.94±0.00)^a^
	(*Z*)-3-hexenyl acetate	(0.11±0.00)^b^	(0.11±0.01)^ab^	(0.13±0.01)^a^	(0.13±0.00)^a^		(0.12±0.00)	(0.12±0.00)	(0.11±0.00)	(0.12±0.00)
	Hexanol	(1.3±0.1)^b^	(1.2±0.1)^b^	(1.5±0.1)^ab^	(1.9±0.2)^a^		(1.53±0.07)	(1.20±0.08)	(1.4±0.1)	(1.7±0.2)
**∑Olive fruit**		**(6.0±2.6)**	**(12.4±1.9)**	**(9.7±0.3)**	**(10.0±1.0)**		**(25.3±5.4)**	**(17.6±3.0)**	**(20.3±3.2)**	**(30.5±0.6)**
Grass	Hexanal	(8.9±0.6)^ab^	(9.9±0.8)^a^	(6.9±1.3)^ab^	(6.4±0.2)^b^		(5.18±0.03)^b^	(9.8±0.9)^a^	(4.2±0.4)^b^	(4.1±0.9)^b^
	(*E*)-2-hexenal	(30.7±3.6)	(28.7±3.6)	(29.2±0.1)	(31.4±1.2)		(23.60±0.01)	(25.9±2.8)	(26.1±1.4)	(21.6±0.6)
	Hexyl acetate	(0.02±0.00)	(0.02±0.01)	(0.02±0.00)	(0.02±0.00)		(0.04±0.00)^a^	(0.03±0.00)^b^	(0.02±0.00)^c^	(0.04±0.00)^a^
	(*Z*)-3-hexenyl acetate	(0.11±0.00)^b^	(0.11±0.01)^ab^	(0.13±0.01)^a^	(0.13±0.00)^a^		(0.12±0.00)	(0.12±0.00)	(0.11±0.00)	(0.12±0.00)
	1-Penten-3-on	(17.6±2.2)	(20.4±3.2)	(15.1±0.5)	(14.7±0.2)		(14.1±0.5)^ab^	(17.3±1.2)^a^	(11.7±0.5)^ab^	(7.6±3.3)^b^
	(*Z*)-3-hexenal	(810±6)^a^	(794±65)^a^	(284±11)^b^	(722.1±16.4)^a^		(151.3±3.2)^c^	(776±50)^a^	(448±32)^b^	(115±3)^c^
	(*Z*)-2-hexen-1-ol	(0.00±0.00)	(0.00±0.00)	(0.00±0.00)	(0.00±0.00)		(0.00±0.00)	(0.00±0.00)	(0.00±0.00)	(0.00±0.00)
	(*E*)-2-hexen-1-ol	(0.11±0.00)^ab^	(0.11±0.01)^b^	(0.12±0.00)^ab^	(0.13±0.01)^a^		(0.29±0.05)^a^	(0.14±0.01)^b^	(0.13±0.02)^b^	(0.27±0.04)^a^
**∑Grass**		**(868±11)^a^**	**(854±71)^a^**	**(336±10)^b^**	**(775±16^a^**		**(194.6±2.7)^c^**	**(829±55)^a^**	**(491±34)^b^**	**(148.7±7.8)^c^**
Apple	Hexanal	(33.4±2.2)	(37.1±2.8)	(25.±4.9)	(24.1±0.6)		(19.4±0.1)^b^	(36.5±3.4)^a^	(15.9±1.4)^b^	(15.2±3.3)^b^
	(*E)*-2-pentenal	(0.25±0.02)	(0.23±0.03)	(0.20±0.01)	(0.27±0.03)		(0.15±0.01)^b^	(0.22±0.02)^a^	(0.16±0.00)^b^	(0.15±0.00)^b^
	(*E)*-2-hexenal	(81.6±9.6)	(76.1±9.4)	(77.4±0.3)	(83.3±3.3)		(62.61±0.03)	(68.8±7.4)	(69.3±3.7)	(57.3±1.7)
	(Z)-3-hexen-1-ol	(0.83±0.02)^b^	(0.81±0.03)^b^	(0.89±0.03)^b^	(1.09±0.06)^a^		(0.25±0.00)^b^	(0.68±0.04)^a^	(0.65±0.04)^a^	(0.26±0.02)^b^
**∑Apple**		**(116.1±7.5)**	**(114.2±6.7)**	**(104.3±5.2)**	**(108.7±2.8)**		**(82.4±0.1)^ab^**	**(106.3±10.8)^a^**	**(86.1±5.2)^ab^**	**(73.0±4.9)^b^**
Sweet	3-Methylbutan-1-al	(1.9±2.8)	(8.1±1.7)	(4.9±0.1)	(4.4±1.4)		(21.3±5.2)	(13.1±2.8)	(15.8±3.0)	(24.8±1.8)
	Hexanal	(357±23)^ab^	(395±30)^a^	(275±52)^ab^	(257±6)^b^		(207±1)^b^	(390±36)^a^	(169.9±15.5)^b^	(163±35)^b^
	Hexyl acetate	(0.02±0.00)	(0.02±0.01)	(0.02±0.00)	(0.02±0.00)		(0.04±0.00)^a^	(0.03±0.00)^b^	(0.02±0.00)^c^	(0.04±0.00)^a^
	(*Z*)-2-pentenol	(0.15±0.03)^b^	(0.71±0.03)^a^	(0.71±0.05)^a^	(0.8±0.1)^a^		(0.20±0.01)^c^	(0.82±0.06)^a^	(0.63±0.03)^b^	(0.94±0.00)^a^
**∑Sweet**		**(359±26)^ab^**	**(404±28)^a^**	**(280±52)^ab^**	**(262±7)^b^**		**(229.±4)^b^**	**(404±39)^a^**	**(186±18)^b^**	**(188±37)^b^**
Bitter	(*E*)-2-hexenal	(86.4±10.2)	(80.±10.0)	(82.1±0.4)	(88.3±3.5)		(66.36±0.03)	(73.0±7.8)	(73.5±3.9)	(60.8±1.8)
	(*E*)-2-penten-1-ol	(0.65±0.04)^ab^	(0.68±0.06)^a^	(0.47±0.03)^b^	(0.56±0.06)^ab^		(0.93±0.02)^a^	(0.76±0.06)^b^	(0.44±0.02)^c^	(0.62±0.00)^b^
	1-Penten-3-on	(1202±154)	(1399±220)	(1034±36)	(1006±11)		(966±38)^ab^	(1187±101)^a^	(804±32)^ab^	(517±226)^b^
	*(E)*-3-hexen-1-ol	(1.08±0.05)	(1.00±0.04)	(0.96±0.04)	(1.2±0.1)		(0.55±0.02)	(0.80±0.03)	(0.73±0.07)	(0.6±0.2)
**∑Bitter**		**(1290±165)**	**(1482±231)**	**(1118±35)**	**(1096±7)**		**(1034±38)^ab^**	**(1262±109)^a^**	**(879±36)^ab^**	**(579±228)^b^**
**Pungent**	1-Penten-3-on	**(1202±154)**	**(1399±221)**	**(1035±36)**	**(1006±11)**		**(966±38)^ab^**	**(1187±101)^a^**	**(804±32)^ab^**	**(517±226)^b^**

The different effect of filtration on the individual volatile compounds in Buža and Istarska bjelica oil samples immediately after filtration is in accordance with the results of Veneziani *et al*. (*11*), who reported that the filtration of different olive oil samples by cellulose filter paper does not result in uniform trends and patterns of the changes of olive oil volatile composition. Different degrees of vegetable water, as well as different amounts and nature of the tissue particles removed by filtration in various monovarietal olive oil types, may be some of the possible causes.

**Fig. 1:**
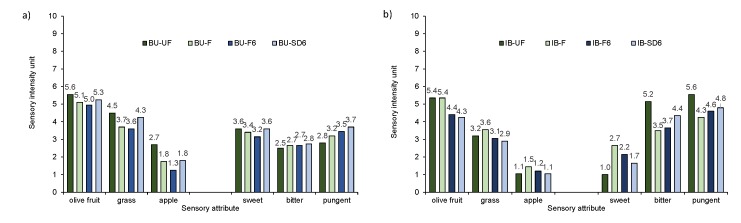
Sensory intensity of the main sensory attributes of oil from cultivars: a) Buža (BU) and b) Istarska bjelica (IB): fresh unfiltered (UF), fresh filtered (F), stored for six months after filtration (F6) and stored for six months after natural sedimentation and decantation (SD6)

According to the results of sensory analysis, Buža and Istarska bjelica unfiltered oil samples included in the study were within stipulated limits of the extra virgin olive oil category (median of fruitiness more than 0, absence of any sensory defects (*20*)), and both filtered monovarietal oil samples remained in the same category of quality, even though some changes in sensory profile of the oil were determined (Fig. 1).

Considering the main odorant series obtained from the odour activity values (Table 4), unfiltered Buža sample had relatively low OAV of olive fruit odour, but multiply higher of grass and apple aromatic series. Similar was observed for unfiltered Istarska bjelica sample, noting that for this cultivar the ratio among the olive fruit, grass and apple odour was more in favour of olive fruit odour characteristics. The reason for the dominant green notes in both cases could be the harvesting at early ripening stage (ripening index RI=2), which is usual for these two cultivars in Croatia. After Buža oil filtration, a slight change, although not significant, was determined: increase in OAV of the olive fruit odorant series and decrease in grass and apple odorant series. The direction of changes in OAVs coincided with the direction of changes determined by sensory analysis, although the increase in intensity (for olive fruit odour) or decrease in intensity (for grass and apple odour) was not quantitatively proportional to the OAV changes (Fig. 1a). The filtration of Istarska bjelica oil did not change OAV or the sensory intensity of olive fruit significantly (Fig. 1b). However, due to filtration of Istarska bjelica oil, multiple increases of grass OAV occurred (primarily due to the increase of (*Z*)-3-hexenal, hexanal and 1-penten-3-on), and an increase at the lower extent for the apple (due to the increase of hexanal and (*Z*)-3-hexen-1-ol). The sensory intensity of these two characteristics also increased, but disproportionately less than an increase of OAV of grass and apple.

**Fig. 2:**
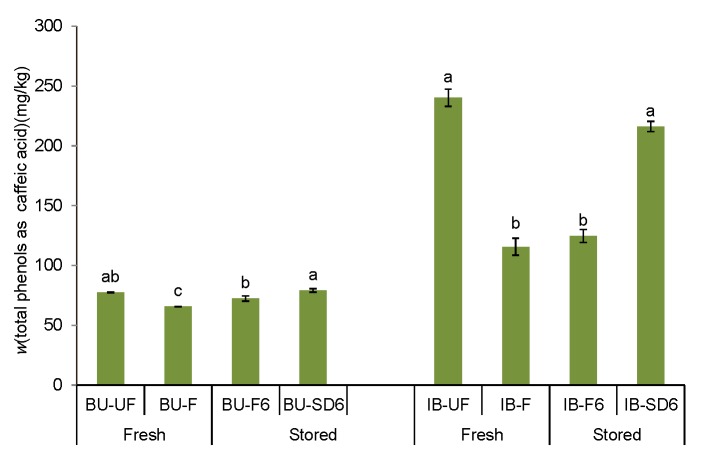
Total phenolic (TP) content in the oil from cultivars Buža (BU) and Istarska bjelica (IB): fresh unfiltered (UF), fresh filtered (F), stored for six months after filtration (F6) and stored for six months after natural sedimentation and decantation (SD6). The results are expressed as the mean value of three independent repetitions of clarifying procedure±SD; the mean values within each cultivar, labelled with different letters, are significantly different (Tukey’s test, p<0.05)

**Fig. 3:**
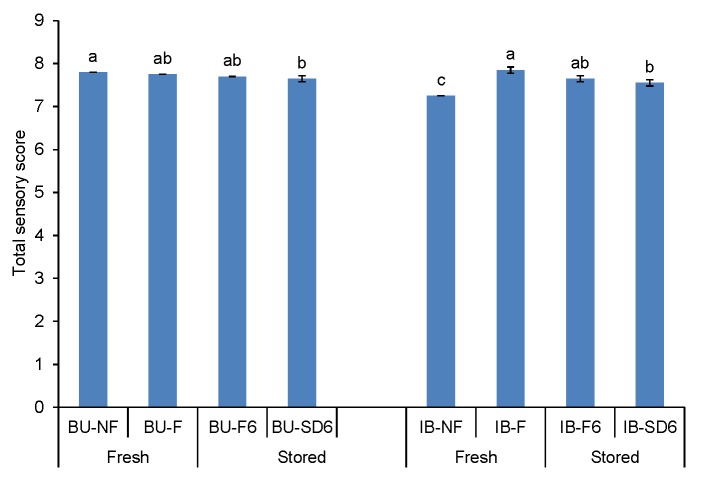
Total sensory score of Buža (BU) and Istarska bjelica (IB): fresh unfiltered (UF), fresh filtered (F), stored for 6 months after filtration (F6) and stored for six months after natural sedimentation and decantation (SD6). The results are expressed as the mean value of three independent repetitions of clarifying procedure±SD, the mean values within each cultivar, labelled with different letters, are significantly different (Tukey’s test, p<0.05)

Particular volatile compounds have been found to have an influence on the sweet, bitter and pungent taste of olive oil (*28*, *32*). In Table 4 it can be noticed that filtration had no significant influence on the OAV of bitter and pungent odorant series in both monovarietal oil samples. As far as sweet note is concerned, an increase was not significant for Buža (13% increase in OAV), but it was significant for Istarska bjelica (an OAV increase of about 75% compared to the unfiltered sample). Comparing OAV changes with the sensory intensity changes for sweet note (Fig. 1), it can be seen that these were similar: panel test showed a negligible decrease in Buža and a significant increase in Istarska bjelica by filtration. However, when it comes to bitter and pungent taste, OAV changes were consistent with the sensory intensity changes by filtration only in Buža oil. In the Istarska bjelica case, despite the lack of significant changes in OAVs, the panel test showed a significant reduction of bitterness intensity (about 1.7% units of intensity) and pungency (about 1.3% units of intensity). As it is evident from Table 5, adjusted Pearson’s *r*_adj_ shows strong correlation between bitter or pungent sensory intensities and mass fraction of total phenols, but weak and even negative correlation with OAV of bitter and pungent taste was determined. Since the taste of VOO is much more influenced by phenolic than volatile compounds (Table 5), differences between the two cultivars considering the relationship between OAV and sensory intensities could be attributed to different effects of filtration on the mass fraction of total phenols (Fig. 2). Unfiltered samples of the two cultivars differed considerably in total phenols (Buža 77 *vs* Istarska bjelica 240 mg/kg), and in sensory intensities of bitter and pungent taste (Buža 2.5 and 2.8 *vs* Istarska bjelica 5.2 and 5.6, respectively). Filtration caused only a slight decrease of total phenols in Buža sample (by about 15%), but a much more important one (by about 52%) in Istarska bjelica sample. Such a filtering effect could be desirable in the oil from Istarska bjelica because it contributed to a more harmonious taste, which was related to an improvement in the total sensory score of the filtered oil compared to the unfiltered sample (Fig. 3). The decrease in the mass fraction of phenols and the intensity of bitterness and pungency could be the result of the retaining of a certain amount of phenols in a filtration aid (*33*-*35*). Veneziani *et al*. (*11*) also found that the amount of phenolic compounds considerably decreases after filtration (in %) of Greek (28.9), Spanish (28.9), Italian (34.5) and Tunisian (54.8) oil through a cellulose filter paper, but not at the same rate in all the studied samples. It seems that the impact of a cellulose filter press system depends on the amounts and nature of the removed fruit particles and vegetable water, which contain most of the phenolic compounds (*36*). Since the probable cause of the decrease of phenolic compounds after filtration is not only the physical retention of substances and particles on the filtering agent, but also the change of the affinity of volatile and phenolic substances to the filtered oil matrix, unequal effects of filtration can also be expected with different oil samples within the same cultivar.

**Table 5:** Strength of correlation between sensory intensity and odour activity value (OAV) of odorant series or mass fraction of total phenols

Sensory intensity	Odorant series	*r*_adj_	Strength level
olive fruit	OAV-olive fruit	0.48	weak to moderate
grass	OAV-grass	0.64	moderate
apple	OAV-apple	0.71	moderate
sweet	OAV-sweet	0.64	moderate
bitter	OAV-bitter	-0.10	weak
bitter	total phenols	0.80	strong
pungent	OAV-pungent	-0.40	weak
pungent	total phenols	0.83	strong
*r*_adj_=adjusted Pearson correlation coefficient			

### Effect of six months of storage of filtered or naturally sedimented/decanted oil

After six months of storage of the filtered oil, a lower loss of volatile compounds was observed in Buža oil than in Istarska bjelica oil (Table 2 and Table 3). In the Buža oil sample the mass fraction of only two volatile compounds (*E*)-2-penten-1-ol and especially (*Z*)-3-hexenal significantly decreased, whereas in Istarska bjelica oil sample such decrease of six volatile compounds, including (*Z*)-3-hexenal, occurred.

In both monovarietal oil samples there was a significant decrease in OAV of the grass odorant series and a slight, although not significant, decrease of apple series (Table 4), which can be associated with a marked reduction of (*Z*)-3-hexenal (contributes to grass odour (*28*)) and hexanal (contributes to grass and apple odour (*28*)) in both oil samples (Table 2 and Table 3). These changes were also noticed in the form of a slight decrease in the intensity of these sensory characteristics (Fig. 1). Regarding the OAV of the olive fruit odorant series in the two monovarietal oil samples, no significant changes were detected. In this case, in Istarska bjelica oil the OAV change (increase in relation to fresh filtered oil by 15%) does not tally with the sensory intensity change of the olive fruit (decrease of 1.0 unit), but in Buža oil, the agreement between the OAV and the sensory intensity was established (Table 4 and Fig. 1).

A reduction of odorant series sweet after six months of storage of filtered oil was determined in both cultivars, although it did not so much affect the intensity reduction of sweetness determined by sensory analysis. Storage of filtered oil did not change significantly the OAV of the odorant series bitter and pungent in both cultivars, which corresponded to the sensory intensity perception of these two taste characteristics (Fig. 1). Stability of the oil taste characteristics of filtered oil samples, bitter and pungent, after six months of storage could also be explained by a relatively slight change in the mass fraction of total phenolic compounds in Buža and by an insignificant change in Istarska bjelica (Fig. 2) since strong correlation between the intensity of these sensory characteristics and total phenols were determined (Table 5). These results are in agreement with previous results that confirmed the stability of total phenol level and taste sensory characteristics of filtered oil during storage (*13*, *36*).

Due to the mild, although mostly unfavourable changes in the sensory intensity of the main odour and taste characteristics of oil, the total sensory scores of fresh filtered and preserved filtered oil samples did not differ significantly (Fig. 3). Overall, these results confirm the presumption that storage of filtered oil reduces the OAV of most of the considered odorant series, but this reduction does not reflect equally on the sensory perception of these properties in the oil from different cultivars. When the effects of the two clarifying methods on the differences in volatile compound mass fractions were compared after six months of storage, it can be said that sedimentation and decantation have advantages over filtration. In contrast to the statistically significant loss of (*Z*)-3-hexenal due to the storage of the filtered Buža sample, there was no statistically significant change in the mass fraction of this compound in the sedimented sample compared to the unfiltered or freshly filtered sample (Table 2). In addition, the mass fraction of (*Z*)-3-hexen-1-ol significantly increased in the headspace of sedimented Buža sample after six months of storage, and because of that, the odour threshold for the active contribution of this compound to apple odour (Table 4) was exceeded. It is unusual that in Istarska bjelica sedimented sample a significant decrease of exactly these two volatiles compared to the filtered samples was detected, while the mass fraction of the other five components (hexyl acetate, octanal, (*Z*)-2-pentenol, (*E*)-2-penten-1-ol and (*E*)-2-hexen-1-ol) increased. Considering the OAV within this group, only (*Z*)-2-pentenol could have a direct contribution to the olive fruit odour.

According to the results of sensory analysis (Fig. 1), filtered and naturally sedimented/decanted oil samples of both cultivars remained in the extra virgin olive oil category after six months of storage. Regarding total sensory score, difference between filtered and sedimented/decanted oil samples after six months of storage was not determined (Fig. 3) but some slight changes of particular sensory attributes were detected (Fig. 1). In Buža oil, a slightly higher intensity of all positive sensory attributes was determined in the sedimented/decanted oil, while in Istarska bjelica oil this trend was observed only in bitter and pungent attributes (Fig. 1), which correlated with the results of total phenols determined in these oil samples (Fig. 2).

Comparing the OAVs of the odorant series olive fruit, grass and apple of sedimented and filtered samples stored for six months (Table 4), the opposite results in two monovarietal olive oil samples were noticed: the higher values determined in Buža oil were lower in Istarska bjelica oil and *vice versa*. Similar to the previously observed pattern, in sedimented oil samples the changes of OAV also coincided more with sensory intensity changes in Buža oil than in Istarska bjelica oil. Observed inconsistency between odorant series approach and sensory analysis (moderate connection in the case of grass and apple and only weak to moderate connection in the case of olive fruit, Table 5) might be due to the synergistic effects of other volatiles that also contribute to these sensory notes of VOOs but were not included in this study.

Considering the sweet odorant series in sedimented and decanted samples, in both cultivars no significant deviations compared to filtered samples were noticed (Table 4), which does not tally with the sensory intensity changes of sweetness (Fig. 1). The values of bitter and pungent odorant series in the sedimented *versus* the filtered samples of Buža oil stored for six months were very similar (deviations of 2 to 3%), which corresponded to similar sensory intensities for these two taste characteristics, as well as the concentration of total phenolic compounds determined in the same oil samples. However, in the case of sedimented Istarska bjelica oil, the OAVs of bitter and pungent odorant series were lower, although not significantly, than of the filtered sample stored for six months (45 to 50%), while sensory intensity of these two characteristic even increased, especially of bitterness. This apparent discrepancy could be attributed primarily to a significantly higher mass fraction of total phenolic compounds in the sedimented Istarska bjelica sample than in the filtered sample after six months of storage (Fig. 2). The observed inconsistency between odorant series approach and sensory analysis in the case of bitter and pungent (Table 5) confirmed as expected that phenolic compounds are more related to the taste of olive oil than volatile compounds.

## CONCLUSIONS

The obtained results confirm that filtration by cellulose filter plate had a different influence on the retention or release of volatile and phenolic compounds in different monovarietal oil samples, despite the fact that there was no difference in the fruit ripening degree or the oil processing method. Considering all the investigated indicators, it can be concluded that the filtration by cellulose filter plate is an acceptable way of clarifying virgin olive oil from Buža cultivar. The present study demonstrated that filtration had a strong effect on the volatile composition, mass fraction of total phenolics and sensory properties of virgin olive oil from Istarska bjelica cultivar. In order to preserve the specific robust sensory characteristics of Istarska bjelica oil, filtration is better to be omitted. Natural sedimentation and decantation of the oil has advantages over oil filtration of both cultivars, especially due to the improved effect on the preservation of the sensory profile and the level of total phenolic compounds. Differences in volatile and phenolic compounds of stored filtered and sedimented and decanted oil samples are additional confirmation of the hypothesis about the essential role of the vegetable water content and the nature and content of tissue particles remaining in the clarified oil. The obtained results can be useful for the optimization of virgin olive oil technology for the production of monovarietal oil with preserved specific and typical sensory characteristics.
